# Effect of Permissive Dehydration on Induction and Decay of Heat Acclimation, and Temperate Exercise Performance

**DOI:** 10.3389/fphys.2016.00564

**Published:** 2016-11-23

**Authors:** Rebecca A. Neal, Heather C. Massey, Michael J. Tipton, John S. Young, Jo Corbett

**Affiliations:** ^1^Extreme Environments Laboratory, Department of Sport and Exercise Sciences, University of PortsmouthPortsmouth, UK; ^2^Young Laboratory, School of Pharmacy and Biomedical Sciences, University of PortsmouthPortsmouth, UK

**Keywords:** thermoregulation, fluid, acclimatization, hydration, hypohydration

## Abstract

**Purpose:** It has been suggested that dehydration is an independent stimulus for heat acclimation (HA), possibly through influencing fluid-regulation mechanisms and increasing plasma volume (PV) expansion. There is also some evidence that HA may be ergogenic in temperate conditions and that this may be linked to PV expansion. We investigated: (i) the influence of dehydration on the time-course of acquisition and decay of HA; (ii) whether dehydration augmented any ergogenic benefits in temperate conditions, particularly those related to PV expansion.

**Methods**: Eight males [VO_2max_: 56.9(7.2) mL·kg^−1^·min^−1^] undertook two HA programmes (balanced cross-over design), once drinking to maintain euhydration (HA_Eu_) and once with restricted fluid-intake (HA_De_). Days 1, 6, 11, and 18 were 60 min exercise-heat stress tests [HST (40°C; 50% RH)], days 2–5 and 7–10 were 90 min, isothermal-strain (*T*_re_ ~ 38.5°C), exercise-heat sessions. Performance parameters [VO_2max_, lactate threshold, efficiency, peak power output (PPO)] were determined pre and post HA by graded exercise test (22°C; 55%RH).

**Results**: During isothermal-strain sessions hypohydration was achieved in HA_De_ and euhydration maintained in HA_Eu_ [average body mass loss −2.71(0.82)% vs. −0.56(0.73)%, *P* < 0.001], but aldosterone concentration, power output, and cardiovascular strain were unaffected by dehydration. HA was evident on day 6 {reduced end-exercise *T*_re_ [−0.30(0.27)°C] and exercise heart rate [−12(15) beats.min^−1^], increased PV [+7.2(6.4)%] and sweat-loss [+0.25(0.22) L.h^−1^], *P* < 0.05} with some further adaptations on day 11 {further reduced end-exercise *T*_re_ [−0.25(0.19)°C] and exercise heart rate [−3(9) beats.min^−1^], *P* < 0.05}. These adaptations were not notably affected by dehydration and were generally maintained 7-days post HA. Performance parameters were unchanged, apart from increased PPO (+16(20) W, irrespective of condition).

**Conclusions**: When thermal-strain is matched, permissive dehydration which induces a mild, transient, hypohydration does not affect the acquisition and decay of HA, or endurance performance parameters. Irrespective of hydration, trained individuals require >5 days to optimize HA.

## Introduction

The heat acclimated phenotype has been extensively described (e.g., Armstrong and Maresh, 1991; Périard et al., 2015) and is characterized by adaptations enabling an individual to better accommodate a given thermal-stressor. Typically, heat acclimation (HA) is acquired by frequently and repeatedly elevating both core (*T*_C_) and skin (*T*_Sk_) temperature (Regan et al., 1996) to a level challenging sudomotor and vasomotor thermoeffector responses for a sufficient duration (Fox et al., 1963). Although passive approaches have sometimes been employed (Beaudin et al., 2009), the increased thermal strain is often achieved through a combination of environmental heat-stress and increased metabolic heat-production through exercise (e.g., Lorenzo et al., 2010; Gibson et al., 2014, 2015; Keiser et al., 2015). More recently, it has been suggested that dehydration, the process of losing fluid and achieving a state of hypohydration (lower-than-normal body water volume), may also represent an important stimulus for facilitating HA (Garrett et al., 2012, 2014; Périard et al., 2015; Akerman et al., 2016), although this may be controversial (Horowitz et al., 1999; Schwimmer et al., 2006) and in contrast to traditional guidelines for maintaining fluid and electrolyte balance (Armstrong and Maresh, 1991; Bergeron et al., 2012).

Dehydration through combined exercise and heat-stress causes hyperosmotic hypovolemia, reducing thermoeffector function [lower sweating and skin blood flow (Sawka, 1992)], and increasing thermal, cardiovascular, and fluid-regulatory strain (Sawka, 1992; Kenefick et al., 2007). Whilst impaired thermoeffector activity might possibly be maladaptive in terms of sudomotor and vasomotor function, the resultant increased tissue-temperature is important; for *T*_C_s between 37.3°C and 38.5°C the magnitude of HA is proportional to the thermal forcing-function (Fox et al., 1963), although increasing *T*_C_ beyond 38.5°C may not confer any additional benefit (Gibson et al., 2014, 2015). Indeed, because dehydration and heat are often inter-linked in their causation and the strain they induce, demarcating their individual effects can be difficult (Akerman et al., 2016). Recent research employing an isothermal strain (target rectal temperature (*T*_re_) = 38.5°C) HA programme suggests that dehydration can provide a thermally-independent adaptation stimulus (Garrett et al., 2014). Restricting fluid ingestion (permissive dehydration) during the five, daily, exercise-heat exposures (90 min·day^−1^) increased plasma aldosterone concentration ([aldo]_p_) over the HA programme, relative to euhydration; this correlated with an increased plasma volume (PV), while increased resting forearm perfusion and reduced exercise heart rate were also observed during a subsequent heat stress test (HST). The adaptations that appear to be most affected by permissive dehydration (e.g., PV expansion and cardiovascular stability) are among the most rapidly acquired during HA (~4–5 days) and also the quickest to decay upon cessation of HA (Williams et al., 1967; Périard et al., 2015). It remains to be established whether permissive dehydration positively influences the adaptive responses to heat over the longer timescales (~10 days) typically necessary to optimize HA (Périard et al., 2015), or whether permissive dehydration affects the retention of the heat acclimated phenotype following HA. Evidence from rodent studies indicates that severe (10% body mass loss) acute hypohydration can adversely affect the longer-term adaptive response to heat (Horowitz et al., 1999; Schwimmer et al., 2006), although the relevance of this work to humans repeatedly dehydrating to a milder hypohydration (<3% body mass loss) over the course of HA is unclear.

The ergogenic potential of HA under more temperate conditions is currently under debate (Minson and Cotter, 2016; Nybo and Lundby, 2016). Lorenzo et al. (2010) demonstrated improved exercise performance in a cool environment (13°C; 30% RH) following a 10-day exercise-heat acclimation programme (40°C; 30% RH) compared to the same training in the cool conditions, possibly related to PV expansion and its influence on VO_2max_ by a Frank-Starling effect. Studies also provide indication that HA elicits improvements in VO_2max_ (Sawka et al., 1985; Lorenzo et al., 2010), exercise economy (Sawka et al., 1983) and lactate threshold (Lorenzo et al., 2010) in temperate conditions; together these are key determinants of endurance performance (Joyner and Coyle, 2008). However, many of these studies have been criticized for inadequate control (Corbett et al., 2014) and this ergogenic effect has not been replicated in recent experiments employing more appropriate controls (Karlsen et al., 2015; Keiser et al., 2015). Moreover, the influence of PV expansion on VO_2max_ depends on the balance between increased cardiac output and the haemodilution effect on O_2_-carrying capacity, which may be unfavorable in an already hypervolemic population. Recently, Keiser et al. (2015) showed no effect of PV expansion on VO_2max_ or exercise performance among a well-trained cohort, whether induced through HA, or by albumin-solution infusion, although there was considerable inter-individual variation. Given that dehydration may augment the hypervolemic aspect of HA (Garrett et al., 2014), understanding the resultant effects on VO_2max_ and exercise performance is important, particularly as these programmes are often used by athletes and individuals undertaking heavy physical work. Interestingly, there is some evidence of an ergogenic effect of short-term HA programmes with permissive dehydration amongst trained individuals in hot (Garrett et al., 2014) and temperate conditions (Neal et al., 2016), but these studies must be interpreted cautiously due to the lack of an appropriate comparison group.

Accordingly, the primary aim of this study was to investigate the influence of permissive dehydration on the time-course and magnitude of the acquisition and decay of HA over a short- and longer-term, using a matched thermal-strain HA programme. An ancillary aim was to investigate the ergogenic potential of HA and specifically to examine whether permissive dehydration augmented any ergogenic effects of HA, particularly those effects related to PV expansion.

## Methods

### Participants

Eight trained male athletes participated in this study which was approved by the University's Ethics Committee [Mean(*SD*) age: 21(3) years; height: 1.81(0.05) m; mass: 77.31(4.88) kg; body fat: 10.0(3.5)%; VO_2max:_ 56.9(7.2) mL·kg^−1^·min^−1^; peak power output (PPO): 338(46) W]. This sample size is consistent with previous work in this area that has identified between-conditions differences in key thermo-physiological indices (Garrett et al., 2014). Participants were all engaged in recreational endurance exercise (running, cycling, triathlon). All participants provided written informed consent.

### Experimental design

**Figure 1:**
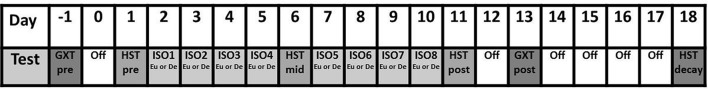
**Experimental protocol for examining the effect of hydration on the adaptive responses to exercise in the heat**. GXT, Graded Exercise Test; HST, Heat Stress Test; ISO, Isothermal strain acclimation session; Eu, Euhydration; De, Dehydration.

A within-participant, balanced cross-over design was employed, with participants undertaking both control [euhydrated heat acclimation (HA_Eu_)] and intervention [permissive dehydration (HA_De_)] HA programmes (target ambient conditions: 40°C; 50% RH). Each HA programme lasted 11-days and consisted of three bouts of exercise at a fixed external work rate [heat stress test (HST)], undertaken on day 1 (HST_pre_), day 6 (HST_mid_), and day 11 (HST_post_), interspersed with eight isothermal heat strain exercise-heat exposures (ISO). The ISO approach was used to induce HA so as to avoid the potential for a dehydration-induced elevation in *T*_re_, which would provide an additional thermal stimulus for adaptation and the HSTs enabled assessment of the induction of short- and longer-term adaptations. A temperate (target ambient conditions: 20°C; 55% RH) graded exercise test (GXT) was completed before (GXT_pre_) and after (GXT_post_) HA for assessment of performance parameters and thermoregulatory responses during temperate exercise. To obtain an index of decay the HST was repeated 1 week after the HA programme (HST_decay_). HA programmes were identical apart from the fluid consumption during ISO, where a regimen was prescribed to either maintain hydration or facilitate dehydration. A minimum 3-month wash-out period was prescribed between HA programmes (see Figure 1). All testing was completed in the UK winter months (November-February) with an average ambient temperature of 2°C during the data collection periods. The average temperature in the 3 months preceding the data collection period was 8°C.

### Experimental procedures

#### Heat stress test

Participants cycled in the hot environment on a calibrated CompuTrainer cycle ergometer (RacerMate Inc., Seattle, Washington, USA) for 60 min at 35% of PPO reached in the GXT (described subsequently). 1.25 L of 3.6% carbohydrate solution (drink temperature 20°C) was ingested to replace fluid losses, divided into five equal boluses (0.25 L) and consumed immediately prior to commencing exercise and every 15 min thereafter. Convective cooling was provided at a rate of 3.5 m·s^−1^; this prevented most participants from reaching the *T*_re_ withdrawal criteria of 40°C, whilst maintaining an acceptably high mean skin temperature (${\bar{\text{T}}}_{\text{Sk}}$) and allowing thermoeffector responses to be assessed.

#### Isothermal heat strain sessions

During each ISO participants exercised in the hot environment on a calibrated CompuTrainer cycle ergometer (RacerMate Inc., Seattle, WA, USA), initially selecting a work rate eliciting a rating of perceived exertion (RPE; Borg, 1982) of 15. This was maintained until *T*_re_ = 38.3°C, at which point external power output was adjusted as appropriate to maintain the target *T*_re_ (38.5°C) and a small amount of convective cooling (~2–3 m·s^−1^) was used to facilitate the exercise component and provide some perceptual benefit whilst maintaining a high ${\bar{\text{T}}}_{\text{Sk}}$. During HA_Eu_ participants consumed 1.75 L of 3.6% carbohydrate-electrolyte fluid (Science In Sport, Nelson, UK) in 0.25 L boluses every 15 min (drink temperature 20°C), including immediately prior to and at the end of each ISO. After the exercise, participants were encouraged to drink *ad libitum* to ensure similar hydration for the following days. Permissive dehydration is defined as purposefully allowing a person to dehydrate through restricting fluid intake (Garrett et al., 2014); during HA_De_ no fluid consumption was permitted during each ISO, or for 10 min after. Thereafter, participants consumed 1.75 L of the aforementioned beverage and were subsequently encouraged to drink *ad libitum* to ensure adequate hydration on arrival the following day. The drinking regimens that we employed were used in a previous study where a clear separation of hydration state was achieved and an influence of permissive dehydration on (short-term) HA was demonstrated (Garrett et al., 2014).

#### Graded exercise test

All GXTs were performed on a Lode Excalibur cycle ergometer (Lode, Groningen, The Netherlands) in a temperate environment. Participants exercised for 20 min at 85 or 110 W, dependent upon the estimated fitness of the participant (fixed within-participant). Thereafter, work-rate was incremented by 25 W every 3 min until blood lactate concentration [Lac] was ≥4 mmol·L^−1^, following which, the participant was given a 5 min break before beginning cycling again at 100 W for 5 min. Work-rate was then increased 25 W·min^−1^ until volitional exhaustion. [Lac] was determined from fingertip capillary blood obtained at the end of each exercise stage (Biosen C-line, EKF Diagnostic, Cardiff, UK). Convective cooling was provided at a rate of 3.5 m·s^−1^.

### General procedures

Participants wore the same clothes (cycling shorts, shoes, socks) on each day, abstained from alcohol throughout the experimental period or caffeine for 12 h before exercise, consumed a similar diet before each test and drank 0.5 L of water 2 h before every attendance. Participants were instructed to maintain their normal high-intensity training (except 24 h before HSTs or GXTs) and replace an equivalent duration of low/moderate training with that completed in the laboratory to maintain usual training volume; this was reiterated throughout the study and verbally verified.

To ensure similar hydration before HSTs and to ascertain the extent to which participants were able to maintain hydration status across the course of each HA regime, urine osmolality was assessed from daily pre-exercise urine samples (Osmometer 3320, Advanced Instruments Inc., Norwood, MA, USA). This equipment was also used to determine plasma osmolality. Nude body mass (dry) was measured pre- and post- each test session (Industrial Electronic Weight Indicator, Model I10, Ohaus Corporation, Parsippany, NJ, USA); body mass changes were used to determine whole-body sweat rate (SR), adjusted for fluid ingested. Ambient conditions were measured by a WBGT logger (Squirrel 1000, Grant Instruments, Cambridge, UK), *T*_re_ by a thermistor (Grant Instruments, Cambridge, UK) self-inserted 15 cm beyond the anal sphincter and cardiac frequency (*f*_C_) by short range telemetry (Polar RS800, Polar Electro, Kempele, Finland). During HSTs and GXTs, skin temperature (*T*_Sk_) was measured using thermistors on the chest, biceps, thigh and calf (Grant Instruments, Cambridge, UK) and local SR [upper-right back (Q-Sweat, WR Medical Electronics, Maplewood, MN, USA)] and forearm skin blood flow (MoorLAB, Moor Instruments, Devon, UK) were recorded. During HSTs expired gases (Douglas bag method), RPE (Borg, 1982), thermal sensation and thermal comfort (Zhang, 2003) were measured at 15 min intervals; a sample of sweat was collected using a custom patch constructed from Parafilm® (Bemis NA, Neenah, WI, USA) for determining sodium concentration [Na^+^] by flame photometry (Corning 400, Essex, UK). During GXTs VO_2_was measured breath-by-breath throughout (Quark B2, COSMED, Rome, Italy).

Immediately before and after HSTs and ISO1 a 10 mL venous blood samples was obtained (K2 EDTA blood collection tubes, Beckton Dickson & Company, Plymouth, UK) from the antecubital vein following 10 min of seated rest for the measurement of hemoglobin concentration [Hb] (201^+^ HemoCue, Sweden) and haematocrit (Hct) (Hawksley, Lancing, UK). Whole blood samples were centrifuged (1500 g for 15 min at 4°C, Heraeus™ Multifuge™ 3 S-R, Thermo Electron Corporation, Germany) and the resultant plasma stored at −80°C for subsequent biochemical analyses using enzyme linked immunosorbent assays for [aldo]_p_ (ELISA Kit #ADI-900-173, Enzo Life Sciences, Exeter, UK) and extracellular heat shock protein 70 concentration (e[HSP70])(Amp'd® HSP70 High Sensitivity ELISA Kit #ENZ-KIT-101, Enzo Life Sciences, Exeter, UK).

### Data analysis

Mean skin temperature was calculated according to Ramanathan (1964) and mean body temperature (${\bar{\text{T}}}_{\text{b}}$) as the weighted mean of *T*_re_ and ${\bar{\text{T}}}_{\text{Sk}}$ according to Parsons (1993). For GXT data the lactate threshold was defined as the power output at [Lac] of 4 mmol·L^−1^, gross mechanical efficiency (GME) was calculated at 185 W (highest work rate below lactate threshold achieved by all participants), and VO_2_max was defined as the highest 15 s VO_2_. Physiological strain index (PSI) was determined according to Moran et al. (1998) and plasma and blood volume shifts were determined according to Dill and Costill (1974). Metabolic heat production (MHP) was calculated according to ISO 8996 Malchaire (2004).

### Statistical analysis

Statistical analyses were undertaken using SPSS (IBM Version. 22, IBM, New York, NY, USA). Significance was set at *P* ≤ 0.05; data are presented as mean(*SD*) unless otherwise stated. Following tests for normality, two-way repeated measures ANOVA was used to analyse the main effects, i.e., changes in responses over time and between condition (HA_Eu_ vs. HA_De_), as well as the interaction effect (i.e., time × condition). The Greenhouse-Geisser statistic was employed to account for violations of sphericity; Bonferroni adjusted Students *t*-tests were used *post-hoc* for analysis of main and interaction effects. *Post-hoc* analysis of significant time effects for ISO sessions were made relative to ISO1 only, with alpha adjusted accordingly. The Wilcoxon sign ranked test was used to analyse ordinal (RPE) data. Relationships between the change in PPO and thermoregulatory parameters were assessed by Pearson's correlation coefficient.

## Results

### Isothermal heat strain sessions

Ambient conditions for ISO sessions were 39.3(0.5)°C, 56.2(5.1)% RH. All participants completed each ISO, in both conditions, with the daily exercise responses to each HA programme summarized in Table 1. A main effect for the influence of condition on mean session body weight loss indicted that hypohydration was achieved in HA_De_ and euhydration maintained in HA_Eu_ [body mass loss −2.71(0.82)% vs. −0.56(0.73)%, *P* < 0.001]. This effect was supported by the plasma osmolality changes within ISO1 whereby a significant condition (*P* = 0.013) and interaction effect were evident (*P* = 0.016), with *post-hoc* analysis indicating that plasma osmolality did not differ between conditions at baseline and was unchanged over HA_Eu_ [Pre = 290(4) vs. Post = 287(4) mOsmo·kg^−1^], but increased over the course of the ISO session for HA_De_ [Pre = 293 (5) vs. Post = 297(7) mOsmo·kg^−1^, *P* = 0.006]. Aldosterone concentration increased over ISO1 (*P* = 0.001), but the extent of any increase was not different between conditions and there was no interaction effect [HA_Eu_Pre = 2651(2700) vs. Post = 5859(4044) pmol·L^−1^; HA_De_ Pre = 2686(2496) vs. Post = 7741(4763) pmol·L^−1^].

**Table 1:** **Mean(*SD*) daily responses during 90 min isothermal strain heat acclimation sessions, with (HA_De_) and without (HA_Eu_) permissive dehydration (*n* = 8)**.

	**ISO1**		**ISO2**		**ISO3**		**ISO4**		**ISO5**		**ISO6**		**ISO7**		**ISO8**		***P*****-value**		
	**HA_Eu_**	**HA_De_**	**HA_Eu_**	**HA_De_**	**HA_Eu_**	**HA_De_**	**HA_Eu_**	**HA_De_**	**HA_Eu_**	**HA_De_**	**HA_Eu_**	**HA_De_**	**HA_Eu_**	**HA_De_**	**HA_Eu_**	**HA_De_**	**Time**	**Condition**	**Interaction**
Time to 38.5°C *T*_*re*_ (min)	29(5)	31(10)	31(6)	28(7)	31(8)	28(8)	31(5)	33(7)	32(8)	29(5)	37(12)	32(6)	39(15)	36(11)	34(11)	32(7)	0.018^*^	0.335	0.812
Average *T*_*re*_ (final 60 min) (°C)	38.68 (0.07)	38.65 (0.18)	38.56 (0.16)	38.62 (0.09)	38.60 (0.08)	38.59 (0.20)	38.60 (0.16)	38.59 (0.08)	38.58 (0.16)	38.60 (0.12)	38.50 (0.19)	38.56 (0.11)	38.43 (0.20)	38.48 (0.11)	38.56 (0.20)	38.57 (0.10)	0.063	0.684	0.899
Average *f*c (beats·min^−1^)	148 (10)	146 (13)	146 (8)	146 (11)	141 (10)	139 (10)	141 (9)	136 (7)	143 (9)	142 (9)	140 (7)	142 (9)	143 (11)	143 (10)	138 (8)	147 (10)	0.019^3^	0.918	0.154
External work rate (W)	80 (19)	70 (22)	105 (19)	88 (20)	90 (22)	81 (25)	93 (18)	92 (19)	97 (26)	91 (17)	97 (28)	97 (19)	109 (28)	98 (16)	108 (29)	106 (18)	<0.001^8^	0.485	0.649
Pre-exercise mass (kg)	76.8 (4.7)	75.9 (4.8)	77.3 (4.3)	76.4 (5.1)	77.4 (4.7)	76.4 (5.2)	77.4 (4.7)	76.4 (5.3)	77.5 (5.0)	76.4 (5.1)	77.3 (5.0)	76.7 (5.1)	77.1 (4.5)	76.5 (5.0)	77.3 (4.3)	76.7 (4.7)	0.186	0.263	0.800
Whole-body SR (L·h^−1^)	1.21 (0.41)	1.18 (0.40)	1.33 (0.31)	1.27 (0.41)	1.33 (0.33)	1.25 (0.34)	1.43 (0.34)	1.29 (0.37)	1.49 (0.35)	1.42 (0.38)	1.48 (0.34)	1.46 (0.35)	1.58 (0.37)	1.56 (0.45)	1.60 (0.40)	1.57 (0.38)	<0.001^4−8^	0.229	0.066
Urine osmolality (mOsmo·kg^−1^)	329 (188)	487 (273)	277 (152)	408 (243)	325 (168)	432 (219)	420 (209)	304 (103)	294 (115)	415 (320)	348 (209)	404 (190)	337 (122)	335 (210)	249 (144)	292 (212)	0.649	0.287	0.442
Body mass loss (%)	−0.26 (0.81)	−2.35 (0.89)	−0.45 (0.69)	−2.51 (0.89)	−0.32 (0.68)	−2.46 (0.75)	−0.54 (0.70)	−2.56 (0.82)	−0.64 (0.72)	−2.80 (0.83)	−0.62 (0.71)	−2.88 (0.77)	−0.78 (0.78)	−3.04 (0.84)	−0.86 (0.82)	−3.09 (0.81)	<0.001^5, 7, 8^	<0.001	0.756

*Significant difference = P < 0.05. Significant post-hoc time effects are relative to ISO1 only and denoted by superscripted letter (^2^ = ISO1 vs. ISO2; ^3^ = ISO1 vs. ISO3; ^4^ = ISO1 vs. ISO4 etc.)*.

* *Post-hoc comparisons not significant relative to ISO1*.

*T_re_, rectal temperature; SR, sweat rate*.

Over the course of each HA programme the time to reach the target *T*_re_ did not differ between conditions and the same elevated average *T*_re_ was maintained over the final 60 min of each session. Average power over the ISO sessions increased, but to a similar extent in both conditions; *post-hoc* analysis identified significant increases from the first day (ISO1) to the final day (ISO8). Conversely, *f*_C_ reduced over time, particularly at ISO3, but again, this did not differ between conditions. Whole-body SR was augmented with HA irrespective of condition, with *post-hoc* comparisons to the initial ISO session indicating that this occurred from ISO4 onwards. Participants managed to maintain a stable pre-exercise body mass and urine osmolality over the course of the intervention, in both conditions, despite an increased sweat rate and temporary hypohydration during HA_De_.

### Heat acclimation

**Figure 2:**
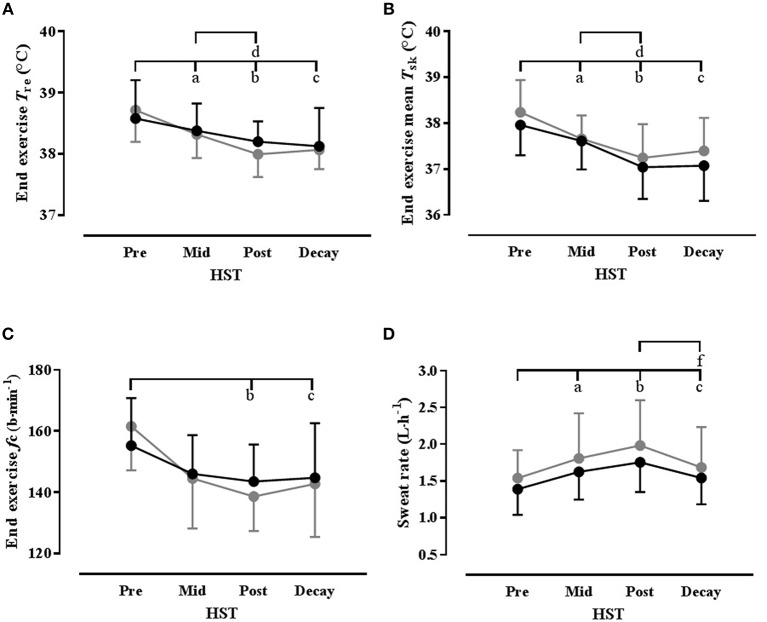
**Select thermophysiological variables showing time course of heat acclimation with (HA_De_: black) and without (HA_Eu_: gray) permissive dehydration as determined from standard heat stress tests (HST)**. Data are mean(*SD*) and *n* = 8 unless otherwise stated. **(A)** End exercise rectal temperature (*T*_re_); **(B)** End exercise mean skin temperature (${\bar{\text{T}}}_{\text{Sk}}$); **(C)** End exercise mean heart rate (*f*_c_); **(D)** Mean HST whole-body sweat rate (*n* = 7). Significant *post-hoc* time effects (*P* < 0.05) are denoted by superscripted letter (^a^ = HST_pre_ vs. HST_mid_; ^b^ = HST_pre_ vs. HST_post_; ^c^ = HST_pre_ vs. HST_decay_; ^d^ = HST_mid_; vs. HST_post_; ^e^ = HST_mid_; vs. HST_decay_; ^f^ = HST_post_ vs. HST_decay_).

The ambient conditions [39.4(0.3)°C, 52.8(2.8)% RH] and the external work rate [Mean 122(14) W] were the same across all HSTs. The thermophysiological, metabolic, biochemical, and perceptual changes over the course of each HA programme, as measured during the HSTs, are summarized in Table 2 (Supplementary Material), with select thermophysiological adaptations shown in Figure 2. A number of main effects for time were identified, with *post-hoc* analysis showing that some HA was evident by HST_mid_, as indicated by significantly reduced thermal strain at rest and during exercise, lower exercise cardiovascular strain, increased whole-body SR and increased blood volume and PV. However, improved thermal comfort and sensation and reduced PSI were only becoming evident at HST_post_ and there were further improvements in a number of thermal parameters from HST_mid_ to HST_post_. These adaptations were well maintained during the decay period with no significant changes in any parameter from HST_post_ to HST_decay_, with the exception of a reduced whole-body SR and RER, whereas MHP was reduced relative to HST_pre_ and suggests improved metabolic efficiency, given that external work rate was unchanged. Plasma aldosterone concentration was not assessed during HST_decay_ but a time effect was evident over the time points assessed (*P* = 0.048). Although the location of this effect could not be identified *post-hoc*, numerically, [aldo]_p_ increased over the HA programme, but this did not differ between conditions and there was no interaction effect.

The only significant differences between HA conditions was for Δ blood volume, which was lower in HA_De_, and also demonstrated a significant time × condition interaction. Although the location of any differences could not be located *post-hoc*, there was a trend for a between-conditions difference in HST_decay_ (*P* = 0.06). An interaction effect was also noted for Δ plasma volume, but again, the location of any differences could not be located *post-hoc*, although numerically, the greatest difference between conditions was also in the decay period.

### Temperate exercise

**Figure 3:**
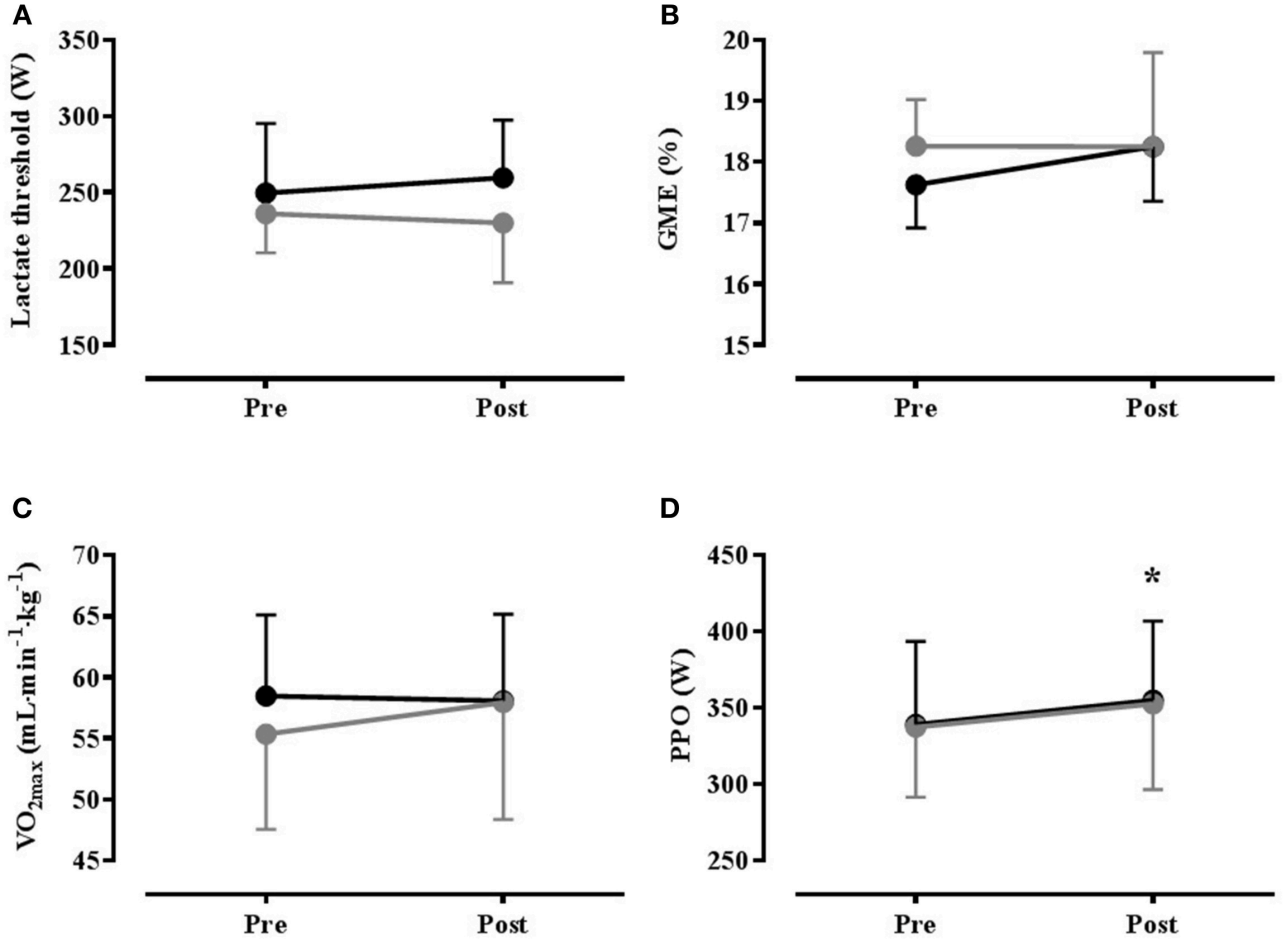
**Mean(*SD*) results from temperate (22°C, 55% RH) graded exercise test performed Pre- and Post- heat acclimation, with (HA_De_: black) and without (HA_Eu_ gray) permissive dehydration (*n* = 8)**. **(A)** Lactate Threshold; **(B)** Gross Mechanical Efficiency (GME); **(C)** Maximal Oxygen Uptake (VO_2max_); **(D)** Peak Power Output (PPO). ^*^ = Significant main effect for time (*P* < 0.05).

Ambient conditions for the GXT were 22.0(0.2)°C, 54.6(5)% RH. Both of the heat acclimation programmes reduced the thermo-physiological burden under temperate conditions, as evidenced by a significant time effect (GXT_pre_ vs. GXT_post_) for resting and exercise *T*_re_ and heart rate, end exercise ${\bar{\text{T}}}_{\text{b}}$ (all reduced), and skin blood flow (increased). The only significant condition effect was for RER, which was higher in HA_Eu_ than HA_De_, but there were no significant interaction effects [see Table 3 (Supplementary Material)]. With regard to parameters related to endurance performance, there were no significant main effects for time or condition, or the time × condition interaction for VO_2max_, lactate threshold or GME (see Figure 3). There was a significant main effect of time on PPO achieved during the GXT (*P* = 0.033), but the condition and interaction effects were not significant (see Figure 3) and the increase in PPO was not correlated with any of the improvements in thermoregulatory function. Likewise, maximum heart rate (*f*_*C*__max_) reached in the GXT was significantly reduced following HA [from 187(7)b·min^−1^ to 183(7) beats·min^−1^ in HA_Eu_ and from 189(10) to 181(9) beats·min^−1^ in HA_De_, *P* = 0.003] but, the condition and interaction effects were, again, not significant.

## Discussion

The main findings of the present study were: (i) there was substantial evidence of adaptation to heat over both the short- and longer-term phases of the present study, but when thermal strain is matched, the time course and magnitude of the acquisition and decay of HA are largely unaffected by permissive dehydration, compared to maintaining euhydration; (ii) permissive dehydration did not notably influence the effect of HA on key parameters related to endurance performance (VO_2max_, LT, GME) and although there was a small ergogenic effect [4.6(5.8)% increased PPO], this was not affected by the drinking regimen.

Our primary finding does not support the suggestion that dehydration provides an additional stimulus for the induction of HA (Garrett et al., 2012, 2014; Périard et al., 2015; Akerman et al., 2016). The data from the short-term phase are somewhat at odds with recent work indicating that dehydrating during 90 min daily exercise-heat stress within a 5-day isothermal HA programme facilitated some aspects of HA (Garrett et al., 2014), but the reason for these discrepant findings is unclear. Aerobic fitness reduces the strain induced by mild hypohydration (Merry et al., 2010) and aerobically fit individuals require a greater stimulus to challenge the fluid-regulatory processes than less fit individuals (Merry et al., 2008). However, the fitness of our participants [VO_2max_ 57(7) mL·kg^−1^·min^−1^; PPO 338(49) W)] was comparable to Garrett et al. (2014) [VO_2max_ 60(7) mL·kg^−1^·min^−1^; PPO 340(30) W] and greater hypohydration lacks ecological validity, could impair some training adaptations (Judelson et al., 2008) and in rodents at least, might impair aspects of the genomic (Schwimmer et al., 2006) and phenotypic (Horowitz et al., 1999) adaptation to heat. A more sustained stimulus might be required to optimize the rebound hypervolemic response (Akerman et al., 2016), but the drinking regimes were virtually identical and earlier, rather than later, carbohydrate-electrolyte fluid replacement is crucial for recovering PV following ~3% body weight loss (Kovacs et al., 2002). Alternatively, because fluid consumption may need to exceed fluid losses by ~50% to restore euhydration in a hypohydrated individual (Shirreffs and Maughan, 1998), the *ad libitum* intake of fluid, electrolyte and protein following the permissive dehydration may have been insufficient to enable any additional hypervolemic adaptation (Kay et al., 2005), but this is not supported by the stable daily baseline body mass and (euhydrated) urine osmolality and while there was some evidence for reduced blood volume change in HA_De_, this appeared to be during the decay, rather than induction, phase.

A clear separation of hydration state was achieved; in HA_Eu_ body mass was maintained consistent with euhydration [−0.56(0.71)% body mass change]; in HA_De_ body mas was reduced [−2.71(0.82)% body mass change] to a degree consistent with hypohydration (Cheuvront et al., 2010; Cheuvront and Kenefick, 2014) and similar to previous studies employing a HA_De_programme (−1.8 to −3.1% average body mass change (Garrett et al., 2012, 2014; Neal et al., 2016). Likewise, baseline plasma osmolality was within the normative range (Cheuvront et al., 2010) and was maintained in HA_Eu_, but increased in HA_De_ to a level consistent with mild dehydration (Cheuvront et al., 2010), although this was not measured in all ISO sessions. Nevertheless, assuming a constant sweating rate, hypohydration (body mass change >−2%) will only have been achieved for the final ~23 min of each ISO and maintained for a further 10 min rest period before fluid consumption, which may have been insufficient to influence the fluid-regulatory mechanisms that are hypothesized to be integral to any effects on HA (Garrett et al., 2012, 2014; Périard et al., 2015; Akerman et al., 2016), although once dehydration is achieved [aldo]_p_ does not appear to further increase with time (Kenefick et al., 2007). Nevertheless, the increased plasma osmolality in HA_De_ did not surpass the threshold 2% increase in osmolality that may be obligatory for compensatory renal water conservation (Cheuvront and Kenefick, 2014) and although [aldo]_p_ was increased by the exercise-heat stress, this was not affected by permissive dehydration, at least within ISO1. Overall, given the substantial similarities in study-design, the reasons for differences between Garrett et al. (2014) and the short-term phase of the present study remain largely unclear. The lack of effect of permissive dehydration over a longer-term HA is, perhaps, less surprising given the modest degree of hypohydration induced, the minimal influence that this likely had on fluid-regulatory mechanisms (Cheuvront and Kenefick, 2014), the rapid time-course over which hematological and cardiovascular adaptations to heat manifest (Armstrong and Maresh, 1991; Périard et al., 2015), and the isothermal strain.

Because some aspects of HA develop rapidly (Périard et al., 2015), there has been considerable interest in short-term HA programmes (Garret et al., 2012, 2014; Neal et al., 2016), particularly for trained individuals who are typically partially heat acclimated and may adapt more rapidly (Périard et al., 2015), as well as for logistical reasons. A recent meta-analysis suggests there is little difference in some aspects of HA over the short and longer-time scales that we studied (Tyler et al., 2016), although few of the studies included repeated measures on the same participants and most employed a controlled work-rate regimen (66%), rather than isothermal-exercise approach (11%), meaning that the adaptation stimulus would have reduced over time. In the present study, which employed an isothermal exercise-heat stress approach, significant hypervolemia, increased whole-body sweat rate and reductions in indices of thermal and cardiovascular strain were evident at HST_mid_, indicating that notable adaptation was achieved within this brief timescale, as others have also demonstrated (Garrett et al., 2012, 2014; Neal et al., 2016). For some indices, such as plasma volume expansion, exercise heart rate and whole body SR, there was no further significant change beyond HST_mid_. In contrast, further reduction in thermal strain, including exercise *T*_re_, ${\bar{\text{T}}}_{\text{Sk},}$ and ${\bar{\text{T}}}_{\text{b}}$, was evident from HST_mid_ to HST_post_, whereas reduced PSI and perceptual benefits (improved thermal comfort and sensation) did not manifest until HST_post_. Taken together, this indicates that the heat acclimated phenotype was not fully developed by HST_mid_. The temporal pattern of adaptation was broadly consistent with the general consensus regarding the time-course of human HA, particularly with respect to the rapid accrual of plasma volume and associated improvement in cardiovascular function (Armstrong and Maresh, 1991; Périard et al., 2015). In contrast, sudomotor adaptations are typically regarded as being slower to develop (Armstrong and Maresh, 1991; Périard et al., 2015), but in the present study whole body sweat rate was unchanged beyond HST_mid_. However, the reducing sweat [Na^+^] will have facilitated sweat evaporation and the progressive reductions in *T*_re_ and ${\bar{\text{T}}}_{\text{b}}$ observed in the HSTs would reduce the thermoafferent sudomotor drive. Moreover, our participants displayed high initial sweating rates, presumably as a consequence of frequent exposure to high endogenous thermal load through their habitual training; fitter individuals have smaller scope for adaptation, but tend to adapt more rapidly than less fit individuals (Périard et al., 2015) and pronounced sudomotor adaption has previously been documented with short-term HA (Neal et al., 2016). Resting [aldo]_*p*_ also increased over the HA regimen, which is in keeping a recent meta-analysis indicating a small effect of HA on resting [aldo]_p_, (Tyler et al., 2016) but e[HSP70] was unchanged following HA. The e[HSP70] response was somewhat surprising since we repeatedly exceeded the proposed endogenous temperature threshold for e[HSP70] release (Gibson et al., 2014), although results from meta-analysis suggests that the effect of HA on e[HSP] is trivial, relative to intracellular [HSP] (Tyler et al., 2016) and basal values may be unchanged during HA (Magalhães et al., 2010). Moreover, the responses could have been blunted by the aerobic training habitually undertaken by our participants and the associated frequent elevations in *T*_C_, which would likely render them partially heat acclimated.

The present study also sought to investigate the extent to which any adaptation to heat was maintained over a 7-day decay period, and whether this was affected by the fluid consumption regimen employed during the HA. Relative to the time-course of induction, the decay in adaptation following HA is poorly documented, but it is generally believed that the hematological and cardiovascular adaptions are among the quickest to decay (Williams et al., 1967; Périard et al., 2015); aspects of the adaptive response most likely to be affected by permissive dehydration (Garrett et al., 2014). Nevertheless, the multitude of approaches used for the induction and assessment of HA and use of limited sample sizes of varying fitness means that there is considerable variation within the published literature regarding the time course of decay of HA. For instance, Williams et al. (1967) reported that, among a group of South African miners who had undertaken a 16 day HA regimen in hot-humid conditions, adaptations in heart rate and mean sweat rate declined by ~50% within 1 week, with a 25% loss in the adaptation in *T*_re_. In contrast, Pandolf et al. (1977) showed little decline in heart rate or *T*_re_ in fit young men up to 18 days after a 9-day dry-heat acclimation regime and Weller et al. (2007) showed little decay in *T*_re_ or heart rate 12 days after completing a 14-day dry-heat acclimation regimen. Indeed, it has been suggested that the retention of HA benefits is superior in aerobically fit individuals and with acclimating to dry heat (Pandolf, 1998). The results of the present study are broadly in keeping with this assertion as there was no significant decay in most of the typical indices of physiological strain HA over the 7-day decay period; although SR and RER were diminished relative to HST_post_, they remained above baseline values and no differences were evident between the drinking conditions. However, these assertions should be tempered by reduced metabolic heat production evident at HST_post_ (discussed subsequently), which occurred despite a fixed external work rate and would have reduced heat-loss requirements during the HST. Moreover, there was a trend for blood volume to decay to a greater extent with HA_De_, but this did not notably influence indices of thermophysiological strain and should be interpreted cautiously given that it was under free-living conditions.

An ancillary aim of the present study was to investigate the ergogenic potential of HA and whether permissive dehydration augmented any ergogenic effects of HA. However, irrespective of drinking regimen, there was no effect of HA on VO_2max_, LT, or GME, but given the similarity in the adaptive response to heat, the lack of between-groups differences is unsurprising. This finding is in contrast to a number of studies that have shown an effect of HA on these parameters (Sawka et al., 1983, 1985; Lorenzo et al., 2010), although these studies have often lacked adequate control and often a simple training effect cannot be excluded (Corbett et al., 2014). The possibility of a training effect was reduced in the present study by the recruitment of competitive athletes, although this may have diminished the adaptation potential due to a ceiling effect, whilst the perception based prescription of work rate during the ISO session and modest hypohydration resulted in similar cardiovascular strain and training stimulus in each group. Although pronounced PV expansion was evident in both drinking conditions, there was no evidence of any change in VO_2max_. This is in contrast to Lorenzo et al. (2010), who demonstrated increased VO_2max_ concomitant with HA induced PV expansion, but is consistent with recent work showing no effect of HA induced PV expansion on VO_2max_ (Karlsen et al., 2015; Keiser et al., 2015). The reason for these equivocal findings is not entirely clear, although in Lorenzo et al. (2010) the relative intensity of training sessions in the heat was higher than for a control group undertaking training under cool conditions and the possibility of an additional training stimulus cannot be excluded. Cardiovascular strain was matched between control and experimental groups in Keiser et al. (2015), although it may have been higher in the experimental group of Karlsen et al. (2015). Alternatively, while the effect of PV expansion on VO_2max_ appears unfavorable at the population level for trained individuals, there appears to be substantial inter-individual variation (Keiser et al., 2015), possibly due to individuality in the balance between increased cardiac output and the haemodilution effect on O_2_-carrying capacity. When pronounced inter-individual variation is combined with relatively small sample sizes, the data may not reflect population characteristics, although at the elite performance level these individual differences may be important.

Although our data from the HST indicate that the O_2_ cost of exercise was diminished 1 week post exercise, this was not evident in the GME data obtained during the GXT. Because the improved economy was specific to performance in a hot environment it could simply represent the effect of reduced thermal strain. Alternatively, a move to a more efficient phenotype has been demonstrated in rodents undergoing prolonged HA (Kodesh et al., 2011); this could explain why this effect had not developed at HST_mid_ or HST_post_. Results from a recent meta-analysis have also concluded that there may be a small effect of HA on GME during exercise in the heat (Tyler et al., 2016), but with the exception of studies lacking appropriate control (Sawka et al., 1983), there appears to be little evidence for an effect of HA on GME in humans under temperate conditions (Karlsen et al., 2015). Nevertheless, a small ergogenic effect was apparent as indicated by a 4.6% increase in PPO achieved at the end of the GXT, irrespective of drinking condition, but the mechanisms underpinning this ergogenic effect are unclear given the lack of change in VO_2max_, LT and GME. The effect of ambient temperature on aerobic exercise is a continuum, with an exponential performance decline at temperatures above ~10°C (Galloway and Maughan, 1997). Although it is clear that HA attenuates the performance decrement in hot environments, it has been hypothesized that the improved thermoregulatory capability with HA should also attenuate the heat-related performance decrement evident under more temperate conditions (Corbett et al., 2014). Indeed significant reductions in thermal-strain were evident in the sub-maximal exercise preceding the GXT, but none of these changes were correlated with the performance improvement, and the *T*_re_ at exercise termination was similar pre vs. post HA, and below the levels associated with impaired performance. Alternatively, we cannot exclude a simple placebo or learning effect on PPO, as we did not include a sham treatment or temperate training group; the primary purpose of the present study was to examine the influence of hydration on HA and performance, rather than the effect of HA *per se*. This assertion is strengthened by our (unpublished) observation of a similar magnitude of improvement in PPO (6.0%) for 8 trained individuals following an identical protocol to the present study, but with all ISO session undertaken with exercise at a matched RPE, under cool conditions (13°C; 60% RH).

In summary, the present study is the first to examine the influence of dehydration on short- and longer-term HA and its subsequent decay, as well as the effect of a longer-term HA regimen with permissive dehydration on key endurance performance parameters. Our data demonstrate that, when thermal strain is matched, the time course and magnitude of the acquisition and decay of HA are largely unaffected by permissive dehydration, compared to maintaining euhydration. Furthermore, neither HA regimen affected VO_2max_, LT, or GME. PPO was increased consistent with a small ergogenic effect of HA, but this was not affected by the drinking regimen and should be interpreted cautiously in the absence of a plausible mechanism. However, it is important to note that no notable negative effects of permissive dehydration were evident either, and traditional guidance to maintain hydration during HA (Armstrong and Maresh, 1991; Bergeron et al., 2012) may be unnecessary when trained individuals commence exercise in a euhydrated state, when thermal strain is matched, and where a transient mild hypohydration is induced.

## Author contributions

RN, HM, MT, JY, and JC were involved in conceptual design, data collection, interpretation, and manuscript preparation. All authors approve the submission of this work and agree to be accountable for all aspects of the work.

## Funding

RN was funded by a joint English Institute of Sport and University of Portsmouth research bursary. Additional project costs were supported by the English Institute of Sport.

### Conflict of interest statement

The authors declare that the research was conducted in the absence of any commercial or financial relationships that could be construed as a potential conflict of interest.
