# Supplementary material for: Effect of Permissive Dehydration on Induction and Decay of Heat Acclimation, and Temperate Exercise Performance
Source: Front Physiol. 2016 Nov 23;7:564. doi: 10.3389/fphys.2016.00564 (PMC5120118; doi:10.3389/fphys.2016.00564)
Supplement: Supplementary file 1 [file Table2.pdf]

Supplementary material

Table 2: Time course of adaptive response to heat acclimation with (HA_{De}), and without (HA_{Eu}), permissive dehydration as determined from standard heat stress test (HST) performed pre, mid, post and 1 week after (decay) heat acclimation. Data are Mean(SD) or Median(range) for non-parametric RPE data. Significant difference=$P \leq 0.05$; n=8 unless otherwise stated. Significant *post-hoc* time effects are denoted by superscripted letter (^{a}=HST_{pre} *vs.* HST_{mid}; ^{b}=HST_{pre} *vs.* HST_{post}; ^{c}=HST_{pre} *vs.* HST_{decay}; ^{d}=HST_{mid}; *vs.* HST_{post}; ^{e}=HST_{mid}; *vs.* HST_{decay}; ^{f}=HST_{post} *vs.* HST_{decay}).

	HST_{pre}		HST_{mid}		HST_{post}		HST_{decay}		*P* value		
	HA_{Eu}	HA_{De}	HA_{Eu}	HA_{De}	HA_{Eu}	HA_{De}	HA_{Eu}	HA_{De}	Time	Condition	Interaction
Thermal											
Resting T_{re} (°C)	37.12(0.23)	37.28(0.50)	36.75(0.18)	37.07(0.46)	36.74(0.39)	36.96(0.43)	36.77(0.43)	37.10(0.47)	0.000 ^{ab}	0.104	0.469
Exercise T_{re} (°C)	37.89(0.30)	37.90(0.49)	37.57(0.24)	37.73(0.41)	37.40(0.20)	37.63(0.30)	37.46(0.31)	37.61(0.48)	0.000 ^{abcd}	0.360	0.489
End exercise T_{re} (°C)	38.72(0.52)	38.58(0.63)	38.32(0.439)	38.38(0.45)	38.00(0.37)	38.20(0.33)	38.07(0.31)	38.13(0.63)	0.001^{abcd}	0.809	0.593
Resting $\overline{T}_{sk}$ (°C)	36.59(0.42)	36.40(0.48)	36.43(0.37)	36.38(0.42)	36.15(0.43)	36.15(0.44)	36.06(0.56)	36.10(0.82)	0.022 ^{acd}	0.768	0.670
Exercise $\overline{T}_{sk}$ (°C)	37.85(0.47)	37.68(0.31)	37.47(0.33)	37.45(0.33)	37.24(0.50)	37.20(0.26)	37.33(0.48)	36.99(0.65)	0.001 ^{abcd}	0.135	0.325
End exercise $\overline{T}_{sk}$ (°C)	38.24(0.70)	37.96(0.66)	37.66(0.51)	37.61(0.62)	37.25(0.73)	37.04(0.69)	37.40(0.72)	37.07(0.77)	0.000 ^{abcd}	0.096	0.851
Resting $\overline{T}_{b}$ (°C)	37.07(0.25)	37.19(0.49)	36.72(0.19)	37.00(0.45)	36.68(0.38)	36.88(0.40)	36.70(0.43)	37.00(0.45)	0.000 ^{abc}	0.137	0.372
Exercise $\overline{T}_{b}$ (°C)	37.88(0.31)	37.88(0.46)	37.56(0.23)	37.70(0.39)	37.39(0.21)	37.58(0.28)	37.45(0.32)	37.55(0.46)	0.000 ^{abcd}	0.427	0.465
End exercise $\overline{T}_{b}$ (°C)	38.67(0.51)	38.52(0.62)	38.25(0.38)	38.30(0.46)	37.92(0.36)	38.09(0.32)	37.99(0.30)	38.02(0.61)	0.000 ^{abcd}	0.890	0.463
Thermoregulatory											
Whole-body SR ($L \cdot hr^{-1}$) $^{n=7}$	1.54(0.38)	1.39(0.35)	1.81(0.61)	1.63(0.38)	1.98(0.62)	1.76(0.40)	1.69(0.55)	1.54(0.36)	0.000 ^{abf}	0.143	0.808
Upper-back SR ($L \cdot m^{2} \cdot h^{-1}$)	0.54(0.16)	0.59(0.17)	0.68(0.20)	0.54(0.16)	0.67(0.23)	0.62(0.15)	0.63(0.25)	0.60(0.20)	0.487	0.663	0.141
Sweat sodium ($mmol \cdot L^{-1}$)	104(23)	123(40)	99(48)	82(31)	88(50)	86(29)	107(47)	107(40)	0.026	0.996	0.194
Skin blood flow (arbitrary units)	279(92)	275(124)	282(63)	287(60)	302(72)	267(102)	258(54)	276(94)	0.863	0.881	0.648
Resting f_c ($beats \cdot min^{-1}$) $^{n=7}$	86(11)	84(9)	80(9)	80(9)	75(11)	77(9)	81(13)	80(11)	0.007 ^{b}	0.855	0.775
Exercise f_c ($beats \cdot min^{-1}$)	150(13)	143(12)	135(11)	135(10)	129(10)	133(11)	135(12)	134(14)	0.000 ^{abc}	0.818	0.153
End exercise f_c ($beats \cdot min^{-1}$)	161(14)	155(16)	144(16)	146(13)	139(11)	143(12)	143(17)	145(18)	0.000 ^{bc}	0.933	0.370
Mean exercise PSI (arbitrary units)	4.8(1.0)	4.3(0.8)	4.0(1.0)	3.9(0.6)	3.7(0.9)	3.9(0.5)	3.8(0.7)	3.6(1.0)	0.000 ^{bc}	0.654	0.338
End exercise PSI (arbitrary units)	7.3(1.5)	6.7(1.7)	5.8(1.4)	6.0(1.1)	4.9(1.2)	5.5(0.7)	5.2(1.1)	5.4(1.8)	0.000 ^{bcd}	0.854	0.298
Δ Plasma volume (%)	0(0)	0(0)	8.1(8.2)	6.3(4.2)	8.1(7.1)	5.5(6.3)	8.5(8.1)	0.3(5.0)	0.002 ^{ab}	0.197	0.050
Δ Blood volume (%)	0(0)	0(0)	5.3(3.3)	3.1(2.3)	5.9(2.5)	3.0(4.0)	7.0(5.6)	0.2(2.4)	0.002 ^{ab}	0.011	0.007
Δ Plasma osmolality (pre *vs.* post exercise) ($mOsmo \cdot kg^{-1}$) $^{n=7}$	-4(6)	-4(7)	-4(9)	-3(7)	-4(4)	-2(4)	-1(7)	1(5)	0.466	0.713	0.939
Biochemical											
Baseline $[aldo]_p$ ($pmol \cdot L^{-1}$)	1728(1213)	1593(1513)	2031(2182)	1965(1776)	2629(2372)	2393(2047)	-	-	0.048	0.521	0.967
Baseline e[HSP70] ($pg \cdot mL^{-1}$) $^{n=6}$	228(137)	223(141)	157(42)	197(121)	160(129)	200(95)	241(138)	237(194)	0.467	0.268	0.845
Metabolic											
External work rate (W)	123(14)	122(14)	123(15)	122(14)	123(17)	120(13)	120(14)	121(13)	0.333	0.786	0.818
Metabolic heat production ($W \cdot kg^{-1}$)	7.5(1.7)	7.4(1.2)	7.4(1.5)	7.5(0.6)	7.3(1.2)	7.2(0.5)	6.8(0.7)	7.0(1.0)	0.186	0.989	0.832
VO_2 ($L \cdot min^{-1}$)	2.10(0.24)	2.06(0.24)	1.99(0.28)	2.01(0.24)	1.97(0.25)	1.95(0.22)	1.89(0.21)	1.91(0.25)	0.007	0.922	0.857
RER	0.92(0.07)	0.90(0.04)	0.94(0.04)	0.94(0.03)	0.98(0.04)	0.93(0.04)	0.91(0.05)	0.91(0.05)	0.011 ^{ef}	0.178	0.176

Perceptual											
RPE	13(5)	13(5)	13(3)	12(3)	12(3)	12(4)	12(4)	11(3)	HAEu: *P*=0.024; HADe: *P*=0.044		
Thermal comfort (cm)	6.1(4.1)	6.8(4.6)	6.7(4.2)	8.1(2.8)	9.0(3.5)	9.4(3.1)	8.8(3.9)	9.6(3.2)	0.000 [bcde]	0.445	0.764
Thermal sensation (cm)	16.5(1.6)	16.1(2.1)	16.3(1.7)	15.3(1.5)	15.3(1.5)	15.0(1.7)	15.0(1.6)	14.4(1.8)	0.000 [bce]	0.244	0.432

T_{re}=rectal temperature; $\overline{T}_{sk}$=mean skin temperature; $\overline{T}_{b}$=mean body temperature; SR=sweat rate; PSI=physiological strain index; $[aldo]_p$=plasma aldosterone concentration; e[HSP70]=plasma heat shock protein 70 concentration; VO_2=rate of oxygen uptake; RER=respiratory exchange ratio; RPE=rating of perceived exertion.
