# Supplementary material for: Effect of Permissive Dehydration on Induction and Decay of Heat Acclimation, and Temperate Exercise Performance
Source: Front Physiol. 2016 Nov 23;7:564. doi: 10.3389/fphys.2016.00564 (PMC5120118; doi:10.3389/fphys.2016.00564)
Supplement: Supplementary file 2 [file Table3.pdf]

Supplementary Material

Table 3: Mean(SD) thermo-physiological responses to exercise in temperate conditions before and after heat acclimation, with (HA_{De}) and without (HA_{Eu}) permissive dehydration (n=8). Data refer to average during 20 minute exercise period, unless otherwise stated. *=significant difference ($P \leq 0.05$).

	Pre-HA		Post-HA		*P* value		
	HA_{Eu}	HA_{De}	HA_{Eu}	HA_{De}	Time	Condition	Interaction
Thermal							
Resting T_{re} (°C)	37.29(0.25)	37.43(0.30)	37.11(0.27)	37.21(0.33)	0.048*	0.128	0.777
Exercise T_{re} (°C)	37.36(0.28)	37.50(0.27)	37.18(0.23)	37.23(0.30)	0.026*	0.188	0.388
End exercise T_{re} (°C)	37.48(0.23)	37.68(0.23)	37.35(0.22)	37.38(0.25)	0.026*	0.110	0.147
Resting $\overline{T}_{sk}$ (°C)	31.54(0.53)	32.06(0.77)	32.10(0.65)	31.88(0.56)	0.325	0.453	0.089
Exercise $\overline{T}_{sk}$ (°C)	29.96(0.58)	30.17(1.15)	29.98(0.53)	29.83(0.67)	0.599	0.901	0.584
End exercise $\overline{T}_{sk}$ (°C)	29.93(0.78)	29.91(1.28)	29.65(0.54)	29.56(0.65)	0.297	0.883	0.910
Resting $\overline{T}_{b}$ (°C)	36.71(0.21)	36.89(0.34)	36.61(0.28)	36.67(0.34)	0.101	0.158	0.415
Exercise $\overline{T}_{b}$ (°C)	36.62(0.26)	36.77(0.34)	36.46(0.24)	36.49(0.33)	0.054	0.277	0.404
End exercise $\overline{T}_{b}$ (°C)	36.72(0.20)	36.90(0.32)	36.58(0.22)	36.60(0.27)	0.047*	0.287	0.196
Thermoregulatory							
Upper-back SR ($L \cdot m^2 \cdot h^{-1}$)	0.15(0.08)	0.17(0.08)	0.16(0.07)	0.12(0.05)	0.402	0.850	0.096
Skin blood flow (arbitrary units)	61(32)	59(20)	66(34)	75(22)	0.044*	0.817	0.374
Resting f_c ($beats \cdot min^{-1}$)	78(12)	79(11)	72(13)	69(13)	0.007*	0.639	0.369
Exercise f_c ($beats \cdot min^{-1}$)	109(7)	114(9)	104(9)	104(6)	0.015*	0487	0.072
Metabolic							
VO_2 ($L \cdot min^{-1}$)	1.85(0.24)	1.95(0.25)	1.89(0.22)	1.83(0.22)	0.091	0.678	0.065
RER	0.91(0.03)	0.88(0.04)	0.91(0.04)	0.89(0.03)	0.447	0.033	0.618

T_{re}=rectal temperature; $\overline{T}_{sk}$=mean skin temperature; $\overline{T}_{b}$=mean body temperature; SR=sweat rate; VO_2=rate of oxygen uptake; RER=respiratory exchange ratio.
